# Case report: Application of targeted NGS for the detection of non-canonical driver variants in MPN

**DOI:** 10.3389/fgene.2023.1198834

**Published:** 2023-06-16

**Authors:** Jin Zhang, Kefeng Shen, Min Xiao, Jinjin Huang, Jin Wang, Yaqin Wang, Zhenya Hong

**Affiliations:** ^1^ Department of Hematology, Tongji Hospital, Tongji Medical College, Huazhong University of Science and Technology, Wuhan, Hubei, China; ^2^ Department of Clinical Immunology, Xijing Hospital, Fourth Military Medical University, Xi'an, China; ^3^ Department of Pediatric Hematology, Tongji Hospital, Tongji Medical College, Huazhong University of Science and Technology, Wuhan, Hubei, China

**Keywords:** triple-negative myeloproliferative neoplasm, next-generation sequencing, JAK2, CALR, MPL, SH2B3

## Abstract

**Background:** JAK2, CALR, and MPL gene mutations are recognized as driver mutations of myeloproliferative neoplasms (MPNs). MPNs without these mutations are called triple-negative (TN) MPNs. Recently, novel mutation loci were continuously discovered using next-generation sequencing (NGS), along with continued discussion and modification of the traditional TN MPN.

**Case presentation:** Novel pathogenic mutations were discovered by targeted NGS in 4 patients who were diagnosed as JAK2 unmutated polycythaemia vera (PV) or TN MPN. Cases 1, 2, and 3 were of patients with PV, essential thrombocythemia (ET), and primary myelofibrosis (PMF); NGS detected JAK2 p.H538_K539delinsQL (uncommon), CALR p.E380Rfs*51 (novel), and MPL p.W515_Q516del (novel) mutations. Case 4 involved a patient with PMF; JAK2, CALR, or MPL mutations were not detected by qPCR or NGS, but a novel mutation SH2B3 p.S337Ffs*3, which is associated with the JAK/STAT signal transduction pathway, was found by NGS.

**Conclusion:** NGS, a more multidimensional and comprehensive gene mutation detection, is required for patients suspected of having MPN to detect non-canonical driver variants and avoid the misdiagnosis of TN MPN. SH2B3 p.S337Ffs*3 can drive MPN occurrence, and SH2B3 mutation may also be a driver mutation of MPN.

## Introduction

Myeloproliferative neoplasms (MPNs) are a group of myeloid tumours characterised by relatively normal differentiation but uncontrolled proliferation of myeloid granulocytes, erythroid cells, and/or megakaryocytes. Classic MPNs include polycythaemia vera (PV, Phenotype MIM number 263300), essential thrombocythemia (ET), and primary myelofibrosis (PMF, Phenotype MIM number 254450) (Arber et al., 2016). The main mechanism of MPN is mutations in genes associated with the JAK/STAT signal transduction pathway, driving excessive proliferation of myeloid cells (Grinfeld et al., 2018). According to the 5th edition of the World Health Organization (WHO) Classification of Haematolymphoid Tumours (Khoury et al., 2022), in addition to blood cell counts and bone marrow biopsy, one of the major diagnostic criteria is the existence of JAK2, CALR, and/or MPL mutations. Approximately 80%–90% of patients with MPN have these driver gene mutations, while the others are patients with triple-negative (TN) MPN and a worse prognosis (Passamonti and Maffioli, 2016; Rumi and Cazzola, 2017).

Currently, there are three commonly used detection methods for MPN gene mutations: fluorescent quantitative PCR (qPCR) (Supplementary Material S1), Sanger sequencing (Supplementary Material S2), and targeted next-generation sequencing (NGS) (Supplementary Material S3). In qPCR, the hybridization probes were designed based on the hotspot mutations of JAK2, CALR, and MPL. qPCR is characterised by its high sensitivity, short detection time, and fair price. However, qPCR also has defects: it requires specific primers, so novel and non-hotspot mutations cannot be detected. Sanger sequencing covers more mutations than qPCR does, but its sensitivity is relatively low (15%–20%), implying that the mutation cannot be detected if the variant allele frequency (VAF) is lower than 15%–20%. More than 98% of mutations can be detected using the high-throughput and high-sensitivity approach of targeted NGS. Both known and novel mutations of MPN are covered with a high sensitivity at a higher cost.

When applied to MPN patients to detect relevant mutations, both qPCR and Sanger sequencing have defects such as incomplete covering loci or low sensitivity. As a result, targeted NGS is especially important for the diagnosis of triple-negative MPN.

## Case description

**FIGURE 1:**
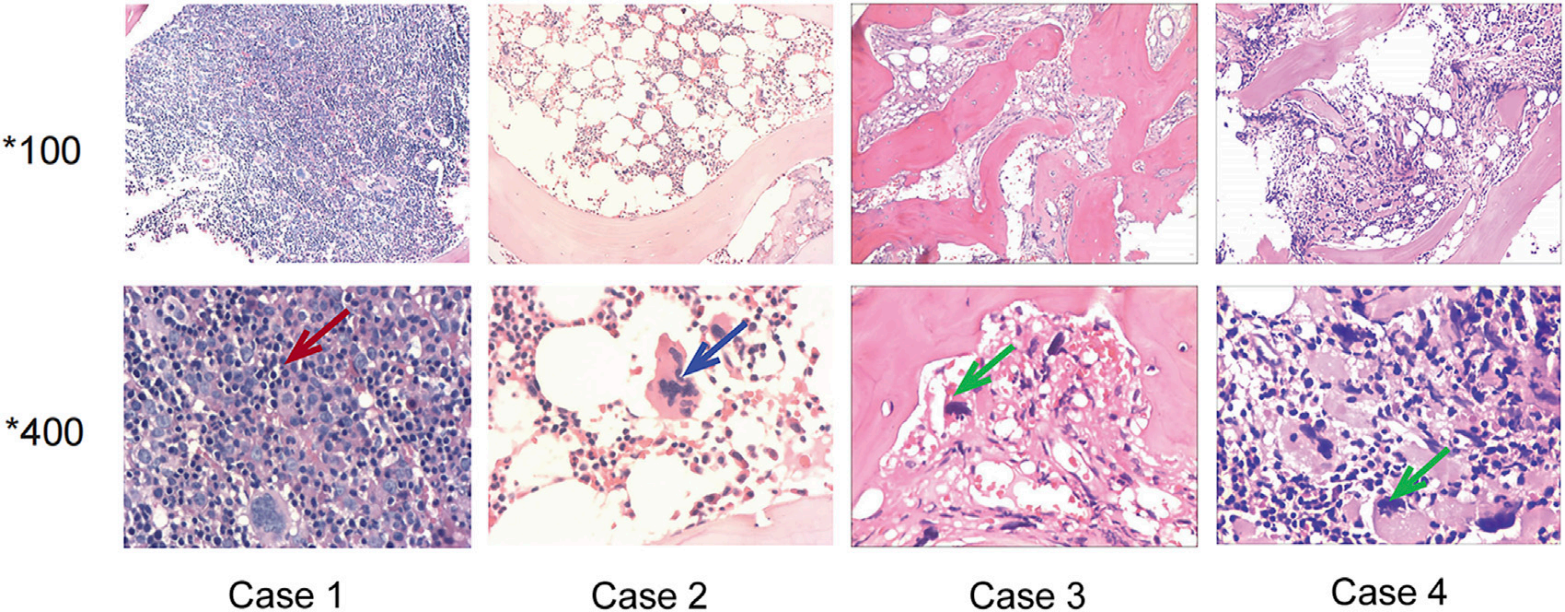
Bone marrow sections of the four patients. Case 1: The red arrow showed erythroblast proliferation, supporting the diagnosis of polycythemia vera. Case 2: The blue arrow showed hyperlobulated megakaryocyte, supporting the diagnosis of essential thrombocythemia. Case 3 and Case 4: The green arrow showed megakaryocytes with atypical and bare nucleus, supporting the diagnosis of primary myelofibrosis.

**FIGURE 2:**
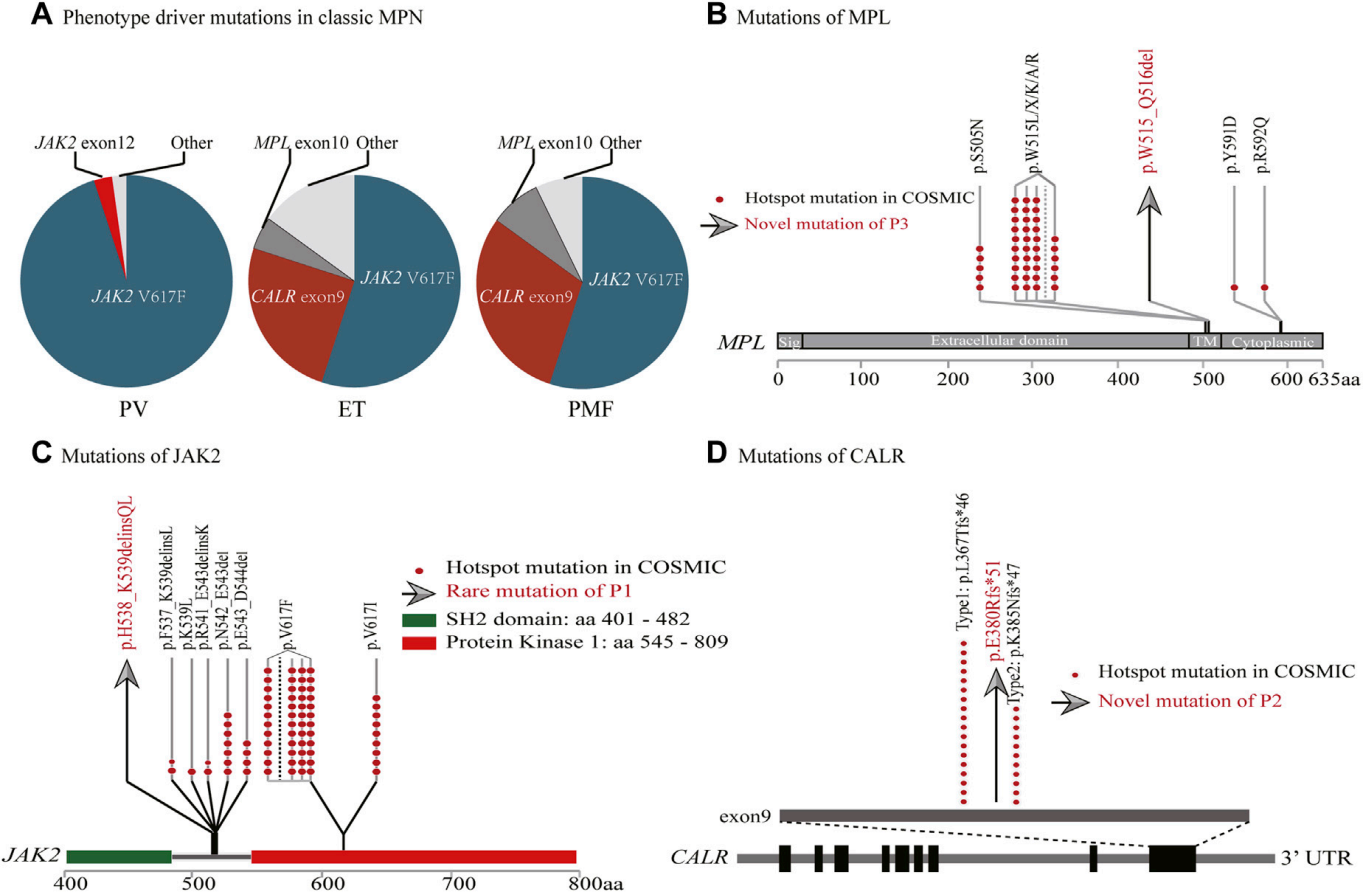
Genomic landscape of myeloproliferative neoplasms. **(A)** Driver gene mutation frequencies. **(B–D)** MPL, JAK2 and CALR gene structures. The red dots represent hotspot mutations in COSMIC. The gray arrows represent rare/novel mutations of the cases (MPL p.W515_Q516del, JAK2 p.H538_K539delinsQL, CALR p.E380Rfs*51).

Case 1 (P1) was of a 30-year-old female patient with PV, who exhibited increased haemoglobin level 4 years ago and then underwent bone marrow aspiration and biopsy in a local hospital. The usual MPN-related gene mutations (Table 1) were not detected using qPCR and the bone marrow biopsy result at the initial diagnosis was lost. She was diagnosed with JAK2 unmutated PV at the same hospital and underwent phlebotomy and oral aspirin therapy. The patient visited our hospital in October 2020, and her haemoglobin level was 198.0 g/L. Bone marrow biopsy was conducted again, and the results confirmed diagnosis of MPN (Table 2; Figure 1). Targeted NGS revealed the presence of JAK2 exon12 mutation (p.H538_K539delinsQL) (Figure 2) with a VAF of 20.9%. This mutation is very rare, and was reported only few times in the COSMIC database before May 2023. COSMIC is the catalogue of somatic mutations in cancer, and is the world’s largest and most comprehensive resource for exploring the impact of somatic mutations in human cancer (https://cancer.sanger.ac.uk/cosmic?genome=37). It was omitted from initial diagnosis because qPCR did not include this locus.

**TABLE 1:** Common MPN driver mutations.

Gene	Mutation type
JAK2	p.V617F
	p.N542_E543del
	p.E543_D544del
	p.K539L
CALR	p.L367fs*46
	p.K385fs*47
MPL	p.W515K/A/L/R/S
	p.S505N

MPN: myeloproliferative neoplasm. del: delete. fs: frame-shift.

**TABLE 2:** Bone marrow examinations.

	P1-PV	P2-ET	P3-PMF	P4-PMF
Films of bone marrow	The percentage of erythrocytes increased, and the central pale area of mature erythrocytes disappeared	Increased platelets	Mature erythrocytes varied in size	Mature erythrocytes varied in size
			Nucleated and tear-drop erythrocytes were found	Nucleated erythrocytes were found
Bone marrow biopsy	Significant hypercellularity (∼90%), proliferation of erythrocytes, slightly proliferation of megakaryocytes, MF-1. MPN to be determined	Hypercellularity (∼50%), hyperlobulated megakaryocytes were found	Hypocellularity (∼30%), and there were megakaryocytes with atypical and bare nuclei. MF-3	Hypocellularity (∼30%), megakaryocytes with atypical, bare nuclei and cloud-like nuclei were found. MF-2
Flow cytometry	No significant abnormalities were found	—	—	—
Karyotype	46, XX	—	46, XX	—
Next-generation sequencing	JAK2 p.H538_K539delinsQL	CALR p.E380Rfs*51	MPL p.W515_Q516del	SH2B3 p.S337Ffs*3
		NFE2 p.R284C	ASXL1 p.G996Sfs*3	
		TET2 p.Q622*	EZH2 p.C590Y	

—: The patient did not accept the test.

Case 2 (P2) was of a 56-year-old female patient with ET. The patient presented with dizziness when she first visited the hospital, after which an increased platelet count was noted. She underwent routine blood tests regularly, and the platelet count increased progressively to 812.0 × 10^9/L. Conditions involving increasing number of reactive platelets, such as in infection, bleeding, or tumours, were excluded. The patient then underwent bone marrow aspiration and biopsy. Aspirated bone marrow films showed clues of MPN (Table 2; Figure 1). MPN-related gene mutations were found to be negative using Sanger sequencing. After 6 months of close monitoring, the platelet count did not decrease. Therefore, targeted NGS was conducted, and CALR p.E380Rfs*51 was detected with a VAF of 12.3% (Figure 2). NFE2 p.R284C with a VAF of 14.9%, and TET2 p.Q622* with a VAF of 9.6% were also detected. CALR p.E380Rfs*51 is a novel driver variant; therefore, any relevant report was not retrieved from the COSMIC. Because of the relatively low sensitivity of Sanger sequencing (15%–20%), the mutation was eliminated at initial diagnosis. Subsequently, the patient was administered interferon therapy. After 2 months of treatment, the platelet count decreased to 761 × 10^9/L. The patient then switched to oral hydroxyurea therapy and maintained a stable platelet count that varied in the range of 560–630 × 10^9/L.

Case 3 (P3) was of a 36-year-old patient with PMF. She was admitted to our hospital because of pleomorphic adenoma, splenomegaly (with a thickness of 6.3 cm), anaemia, increased leukocyte count, platelet count, and lactate dehydrogenase (LDH) level (954 U/L). Additionally, the patient had constitutional symptoms of night sweats and weight loss. She underwent bone marrow aspiration and biopsy. Peripheral blood films and bone marrow tissues confirmed the diagnosis of MPN (Table 2; Figure 1). Common MPN-related mutations were confirmed to be negative using qPCR. Targeted NGS was performed, and the MPL p.W515_Q516del (Figure 2) mutation with a VAF of 68.9% was identified. ASXL1 p.G996Sfs*3 with a VAF of 46.7%, and EZH2 p.C590Y with a VAF of 48.3% were also identified. MPL p.W515_Q516del is a novel driver variant, and has not been reported in the COSMIC. qPCR did not cover this locus; therefore, this mutation was omitted. After 3 months of treatment with JAK2 inhibitor, the platelet count decreased to normal, constitutional symptoms disappeared, and the spleen shrank by more than 50%.

Case 4 (P4) was of a 65-year-old patient with PMF who was diagnosed with triple-negative MPN 3 years ago. He presented to the hospital with fatigue and dyspnoea lasting 8 months. The patient exhibited constitutional symptoms including significant weight loss of 7.5 kg in 20 days. After admission to the hospital, anaemia, increased LDH level (998 U/L), and splenomegaly (with a thickness of 4.7 cm) were observed. Peripheral blood smears and bone marrow tissues revealed the diagnosis the MPN (Table 2; Figure 1). Both qPCR and targeted NGS did not detect JAK2, CALR, or MPL mutations; however, the SH2B3 p.S337Ffs*3 with a VAF of 37.3%, which is associated with JAK/STAT signal transduction pathway, was detected by NGS. The mutation is novel; therefore, any relevant report was not retrieved from the COSMIC. After receiving stimulus to erythrocytes and support treatment, the patient voluntarily left the hospital.

## Discussion

JAK2 gene is located on chromosome 9p24 and encodes one of the four non-receptor tyrosine kinases of the Janus kinase (JAK) family which is involved in the JAK/STAT signal transduction pathway. Its abnormalities, such as those due to mutations, loss of heterozygosity (LOH) on the short arm of chromosome 9 (9p LOH), and copy amplification, are common in haematologic tumours, inducing consistent activation of the JAK/STAT pathway and eventual incidence and progression of disease (Kralovics et al., 2005). JAK2 mutations can be found in approximately 98% of PV, 50%–60% of ET, and 50%–60% PMF cases. Common JAK2 mutations are p.V617F (in exon14), whereas a minor number are due to deletion/insertion in exon12 which is clustered in amino acids 535–547, such as p.N542_E543del. Mutations in exon12 can be found in approximately 1%–3% of PV patients, and are very rare in ET and PMF patients (Scott et al., 2007). P1 (a PV patient) possessed JAK2 p.H538_K539delinsQL mutation, and the incomplete covering sites of qPCR led to the omission. We then performed pathogenicity prediction analysis using Mutation taster, a pathogenicity prediction tool; JAK2 p.H538_K539delinsQL is predicted to be deleterious (Schwarz et al., 2014).

Patients who have typical clinical patterns of PV but lack JAK2 V617F or JAK2 exon 12 mutations are extremely rare (Rumi and Cazzola, 2017). Therefore, when facing these patients, clinicians should perform the differential diagnosis again. If the diagnosis is confirmed, then NGS should be employed to determine whether the patient has an unusual JAK2 mutation.

CALR gene is located on chromosome 19p13 and encodes for a multifunctional calreticulin residing in the endoplasmic reticulum and nucleus. Calreticulin cooperates with other molecules to maintain calcium ion homeostasis, and regulate cell proliferation, apoptosis, and migration. CALR mutations mainly include deletion/insertion in exon9, with type 1 (p.L367Tfs*46), and type 2 (p.K385Nfs*47) comprising about 84.7%, while other types are relatively rare. The frame-shift mutation of CALR exon9 conduces a new C-terminal, activating MPL and JAK/STAT pathways that are vital pathogenic factors of MPN. CALR mutations can be found in 20%–30% of ET and 30%–40% of PMF cases, whereas they are rare in PV (Imai et al., 2017; How et al., 2019). The CALR p.E380Rfs*51 of P2 (a ET patient) is a newly occurring type with a low VAF value; as a result, it was omitted by Sanger sequencing. This mutation is predicted to be deleterious using Mutation taster.

Triple-negative ET patients seemed to have better overall survival than driver gene mutated patients. Tefferi A et al. reported that TN ET patients displayed lower incidence of thrombosis compared with JAK2-mutated cases (Tefferi et al., 2014a). In another study, TN ET patients had significantly lower symptom load, and slightly longer survival than mutated cases (Santoro et al., 2022). Considering these differences, it is necessary to apply targeted NGS to detect non-canonical driver variants.

MPL, which is located on chromosome 1p34, encodes thrombopoietin receptor protein (TpoR) and participates in the activation of JAK/STAT signal transduction pathway. MPL mutations are clustered at exon10; W515 and S505 locus missense mutations are the most frequent types, and other types (such as S204, Y591, and R592) are occasionally found. MPL mutations can result in over-activation of JAK/STAT, promoting the occurrence of tumours. MPL mutations can be found in approximately 3%–5% of ET and 5%–10% of PMF cases, while they are rarely seen in PV (Cabagnols et al., 2016; Milosevic Feenstra et al., 2016). The MPL p.W515_Q516del mutation of P3 (a PMF patient) is also a newly occurring mutation type. It was excluded because qPCR did not cover the locus, either. The analysis by Mutation taster showed this mutation leads to amino acid sequence change, whereas it may be benign.

Triple-negative PMF is an aggressive myeloid neoplasm with significantly worse survival than driver gene mutated cases. A study examined the long-term disease outcomes in 428 patients with PMF (Tefferi et al., 2014b). They discovered that TN PMF patients displayed significantly worse survival (median, 2.3 years), compared to that of CALR (15.9 years), JAK2 (5.9 years), or MPL (9.9 years) mutated patients. Leukaemia-free survival (LFS) in PMF was significantly worse in the presence of triple-negative mutational status, either. Therefore, it is important to distinguish between real and pseudo TN PMF. In our study, P3 responded well to the JAK2 inhibitor, also implying that the patient may not be a real TN PMF patient.

To sum up, targeted NGS should be applied to detect non-canonical and low burden driver variants in MPN, such as JAK2 p.H538_K539delinsQL, CALR p.E380Rfs*51, and MPL p.W515_Q516del, to avoid the misdiagnosis of JAK2 unmutated PV and TN MPN.

SH2B3 is located on chromosome 12q24, encoding the LNK protein which can inhibit JAK/STAT signal transduction pathway by directly binding to JAK2 (Tong et al., 2005; Bersenev et al., 2008). SH2B3 mutations occurred in 5%–7% MPN patients, and the majority are frame-shift-truncated and non-sense mutations in the PH and SH2 domains, resulting in loss of function (Lasho et al., 2010; Maslah et al., 2017). SH2B3 p.S337Ffs*3 of P4 leads to the early emergence of stop codon, and shortens the length of mRNA significantly. Consequently, nonsense-mediated mRNA decay occurs, and LNK protein cannot be synthesized. The mutation is clearly deleterious. In summary, SH2B3 mutation is able to relieve the reverse regulating effect of LNK, and over-activate JAK/STAT. As mentioned above, MPN is characterized by elevated JAK/STAT activity, thus SH2B3 mutation may also be a driver mutation of MPN.

In summary, P1 was formerly misdiagnosed as JAK2 unmutated PV, P2 and P3 were formerly misdiagnosed as triple-negative MPN, but all later detected to contain non-canonical driver mutations by targeted NGS. P4 did not have JAK2, CALR or MPL mutations, but was detected to contain a mutation involved in the negative regulation of JAK/STAT pathway by targeted NGS. All these cases imply the important role of NGS in detecting MPN-related mutations. qPCR does not cover the complete loci and the sensitivity of Sanger sequencing is relatively low, consequently, about 5%–10% mutations can be eliminated. NGS can detect both canonical and non-canonical mutations, and the sensitivity of NGS is higher than that of qPCR and Sanger sequencing. Notably, NGS can detect JAK2, CALR, and MPL mutations as well as other mutations (such as SH2B3 and NFE2) that are associated with the JAK/STAT pathway and haematopoiesis regulation, favouring the discovery of new driver mutations in MPN (Jeromin et al., 2016; Zoi and Cross, 2017).

## Conclusion

NGS, a more multidimensional and comprehensive gene mutation detection, is required for patients suspected of having MPN to detect non-canonical driver variants and avoid the misdiagnosis of TN MPN. SH2B3 p.S337Ffs*3 can drive MPN occurrence, and SH2B3 mutation may also be a driver mutation of MPN.

## Data Availability

The original contributions presented in the study are included in the article/Supplementary Materials, further inquiries can be directed to the corresponding authors.
