# Supplementary material for: Case report: Application of targeted NGS for the detection of non-canonical driver variants in MPN
Source: Front Genet. 2023 Jun 16;14:1198834. doi: 10.3389/fgene.2023.1198834 (PMC10313112; doi:10.3389/fgene.2023.1198834)
Supplement: Supplementary file 1 [file Table1.docx]

Table 1: qPCR panel for MPN

| Gene | Covering mutations |
| --- | --- |
| *JAK2* exon14 | V617F |
| *JAK2* exon12 | N542_E543del, E543_D544del, K539L |
| *CALR* exon9 | L367fs*46, K385fs*47 |
| *MPL* exon10 | W515K/A/L/R/S, S505N |
