# Supplementary material for: Case report: Application of targeted NGS for the detection of non-canonical driver variants in MPN
Source: Front Genet. 2023 Jun 16;14:1198834. doi: 10.3389/fgene.2023.1198834 (PMC10313112; doi:10.3389/fgene.2023.1198834)
Supplement: Supplementary file 2 [file Table2.docx]

Table 2: Sanger sequencing panel for MPN

| Gene | Covering region | Forward primer | Reverse primer |
| --- | --- | --- | --- |
| *JAK2* exon14 | codons 593-621 | 5′-GGGGTTTCCTCAGAACGTTGA-3′ | 5′-CACCTAGCTGTGATCCTGAAACT-3′ |
| *JAK2* exon12 | codons 505-547 | 5′-TCAAAGTTCAATGAGTTGACCCC-3′ | 5′-CTTGAGAACTTGGGAGTTGCG-3′ |
| *CALR* exon9 | codons 350-390 | 5′-CACCAACGATGAGGCATACG-3′ | 5′-CAAAATCCACCCCAAATCCGAA-3′ |
| *MPL* exon10 | codons 502-522 | 5′-CCGAAGTCTGACCCTTTTTGTC-3′ | 5′-TGTTAGAGTGTAAGGAGCCGC-3′ |
