# Supplementary material for: Case report: Application of targeted NGS for the detection of non-canonical driver variants in MPN
Source: Front Genet. 2023 Jun 16;14:1198834. doi: 10.3389/fgene.2023.1198834 (PMC10313112; doi:10.3389/fgene.2023.1198834)
Supplement: Supplementary file 3 [file Table3.docx]

**Method of NGS:**

Targeted next-generation sequencing (NGS) of a panel including 80 genes involved in myeloid neoplasms was performed (Table 1). The library was prepared with AmpliSeq™ Library PLUS for Illumina, and paired‐end sequencing was performed on NextSeq™ 550 platform (Illumina, San Diego, USA). The alignment and variant calling were performed using the DNA Amplicon workflow with default parameters on BaseSpace Sequence Hub (Illumina). Generated variants were further annotated using Annovar.

Variant filtering was performed by the following cascade of steps: 1) select exon nonsynonymous or splice donor site variants; 2) exclude variants with population frequency > 0.001 in the gnomAD database unless the variant was included as a somatic variant of hematopoietic neoplasms in the COSMIC database; 3) exclude variants present in our in-house curated blacklist; 4) exclude variants with Q score < 30 or read depth < 20x or variant allele fraction (VAF) < 1%.

Table 3. Gene list in targeted NGS of 80 genes, covering mutational hotspot regions or whole coding sequences (CDS) within 5 intronic base pairs around exons.

| **Gene list** |  |  |  |  |  |  |
| --- | --- | --- | --- | --- | --- | --- |
| *ABL1* | *ALPP* | *ASXL1* | *ASXL2* | *BCL7A* | *BCOR* | *BCORL1* |
| *CALR* | *CBL* | *CBLB* | *CCND2* | *CDKN2A* | *CEBPA* | *CSF1R* |
| *CSF3R* | *CSNK1A1* | *CTCF* | *DNMT3A* | *ETNK1* | *ETV6* | *EZH2* |
| *FAM5C* | *FAT1* | *FBXW7* | *FGFR1* | *FGFR3* | *FLT3* | *FOXO1* |
| *GATA1* | *GATA2* | *GNAS* | *GNB1* | *HRAS* | *IDH1* | *IDH2* |
| *IKZF1* | *JAK1* | *JAK2* | *JAK3* | *KDM6A* | *KIT* | *KRAS* |
| *LTB* | *LUC7L2* | *MPL* | *NF1* | *NFE2* | *NPM1* | *NRAS* |
| *PDGFRA* | *PHF6* | *PIGA* | *PPM1D* | *PRPF40B* | *PTPN11* | *RAD21* |
| *RECQL4* | *RET* | *RIT1* | *RUNX1* | *SETBP1* | *SF1* | *SF3A1* |
| *SF3B1* | *SH2B3* | *SLC34A2* | *SMC1A* | *SMC3* | *SOCS1* | *SRSF2* |
| *STAG2* | *STAT3* | *STAT5B* | *TET1* | *TET2* | *TP53* | *U2AF1* |
| *U2AF2* | *WT1* | *ZRSR2* |  |  |  |  |
